# Cascade exciton-pumping engines with manipulated speed and efficiency in light-harvesting porous π-network films

**DOI:** 10.1038/srep08867

**Published:** 2015-03-09

**Authors:** Cheng Gu, Ning Huang, Fei Xu, Jia Gao, Donglin Jiang

**Affiliations:** 1 Department of Materials Molecular Science, Institute for Molecular Science, National Institutes of Natural Sciences, 5-1 Higashiyama, Myodaiji, Okazaki 444-8787, Japan

## Abstract

Light-harvesting antennae are the machinery for exciton pumping in natural photosynthesis, whereas cascade energy transfer through chlorophyll is key to long-distance, efficient energy transduction. Numerous artificial antennae have been developed. However, they are limited in their cascade energy-transfer abilities because of a lack of control over complex chromophore aggregation processes, which has impeded their advancement. Here we report a viable approach for addressing this issue by using a light-harvesting porous polymer film in which a three-dimensional π-network serves as the antenna and micropores segregate multiple dyes to prevent aggregation. Cascade energy-transfer engines are integrated into the films; the rate and efficiency of the energy-funneling engines are precisely manipulated by tailoring the dye components and contents. The nanofilms allow accurate and versatile luminescence engineering, resulting in the production of thirty emission hues, including blue, green, red and white. This advance may open new pathways for realising photosynthesis and photoenergy conversion.

Light harvesting initiates photosynthetic processes, whereas antennae serve as a photon engine that pump excitons to a reaction center, where photoenergy is transformed into chemical energy^1^,^2^,^3^. Nature produces light-harvesting antennae by organising multiple chlorophyll units into ring or wheel-like structures, and these structures constitute a vectorial cascade energy-transfer diagram that enables the long-distance yet rapid and efficient pumping of excitons before annihilation^1^,^2^,^3^. Inspired by the intricate mechanism observed in nature, artificial antenna systems for pumping excitons have long been pursued^4^,^5^,^6^, given the promise of applications in solar energy storage and conversion^7^, light amplification^8^, sensing^9^ and luminescent materials^10^. In particular, the development of controlled cascade energy transfer may lead to the next generation of optoelectronic materials for energy transduction and conversion. Although linear polymers^11^ and dendrimers^12^ can covalently link energy-donor, relay and acceptor units into cascade energy-transfer systems to make luminescent polymers, the compositions and contents of these building blocks cannot be freely tailored; to achieve optimised structures for these systems, tedious synthesis is unavoidable. Moreover, the aggregation of polymer chains and the folding of dendritic branches result in complicated interactions among components. These drawbacks must be addressed before custom luminescent materials with easily tunable luminescence, improved efficiency and low cost can be produced. Small chromophores^13^ have be developed for light harvesting via supramolecular assemblies; however, control over the aggregates of multiple components is still semi-empirical, whereas specific interactions and the delicate conditions that are required for maintaining the desired assembled states largely limit the scope of their applications. Porous materials^14^,^15^,^16^,^17^,^18^,^19^,^20^ have been utilised to build up supramolecular host-guest systems for light harvesting and energy transfer. Nevertheless, these systems are limited to bi-component energy donor and acceptor systems; a porous material that can be used to achieve controlled cascade energy transfer is unprecedented.

Porous organic polymers (POPs)^21^,^22^,^23^,^24^,^25^, a class of cross-linked, amorphous polymers with permanent porosity, have emerged as a new platform for the construction of functional and stable porous materials. The development of various chemical reactions, building blocks and synthetic methods has generated a wide range of POPs with specific structures and outstanding properties, driving the rapid growth of the field. POPs have exhibited tremendous potential for adsorption and separation^26^,^27^,^28^,^29^,^30^, catalysis^31^,^32^,^33^, energy storage^34^,^35^ and optoelectronics^36^,^37^,^38^,^39^. However, POPs are usually obtained as unproceessable powders. Recently, we established an electrochemical approach for the preparation of POP films, providing a new platform for functional exploration^40^,^41^,^42^.

Herein, we report light-harvesting POP films into which controllable cascade energy transfer is integrated, leading to a new generation of customisable luminescent materials that are engineered to be easily tailored. The POP films are synthesized with a controlled thickness via the previously reported electrochemical method and emit bright blue luminescence. The three-dimensional π-network drastically enhances the interface of energy transfer to relay and acceptor dyes, whereas the nanopores within the film permit segregated molecular docking of multiple laser dyes with desired properties and in specific proportions. Mechanistic studies reveal that the light-harvesting films allow the integration of a cascade energy-transfer engine that functions in a vectorial and highly efficient manner and enables total control over the speed and efficiency of the energy transfer through tuning of the distribution and types of dye introduced. We show the accuracy and versatility of luminescence engineering with the POP films in producing thirty different luminescence hues, including blue, green, red and white (Figure 1). By virtue of the antenna effect and dye confinement, the films exhibit considerably amplified luminescence and gain exceptional quantum efficiencies. Replacement of the dyes with redox-active components may lead to application of the present technology for highly efficient photosynthesis and photoenergy conversion.

## Results and Discussion

### POP film synthesis and characterisations

**Figure 1:**
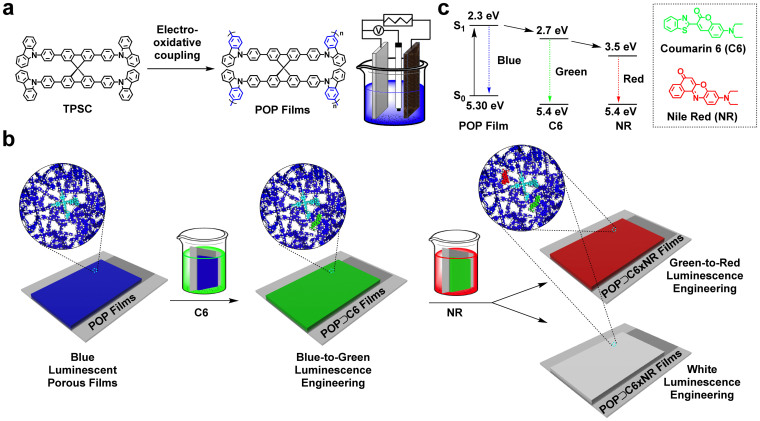
Synthetic scheme and cascade energy transfer diagram. (a), Schematic representation of the synthesis of POP films by the electro-oxidative coupling reaction. Inset: cell setup for electro-oxidative coupling for the preparation of POP thin films on ITO working electrode. (b), Graphical protocol for the preparation of exciton-pumping films by dipping the films into the corresponding laser dye solutions of coumarin 6 and Nile red followed by rinsing. Control of the coumarin 6 and Nile red contents in the films allows elaborate engineering of thirty luminescence colours ranging from blue to green to red and finally white. Inset: Enlarged circles show porous structures of the frameworks (blue: POP framework; sky blue: highlight of one TSPC unit; green: coumarin 6; red: nile red). (c), Diagram of cascade energy transfer from the POP film to coumarin 6 and to Nile red. Inset: Chemical structures of coumarin 6 and Nile red.

To fabricate light-harvesting POP films, we employed orthogonal 2,2′,7,7′-tetraphenyl-9,9′-spirobifluorene as the focal core; this compound strongly absorbs in the ultraviolet region and emits highly efficient deep-blue luminescence, whereas the four *N*-substituted carbazole groups at its periphery serve as electroactive modules for polymerisation (Figure 1a, TPSC; Supporting Information). *N*-Substituted carbazole units, upon electro-oxidative reaction, form radical cation species that are highly active toward coupling with each other and yield dimers in near-unity efficiency without the generation of other oligomers^43^,^44^,^45^,^46^. The POP thin films were formed on an ITO electrode via multicycled cyclic voltammetry (CV) of TPSC. During the first cycle of the positive CV scan, the oxidative potentials of carbazole and spirobifluorene were 0.95 and 1.15 V, respectively (Figure S1a)^47^,^48^,^49^. Two reductive peaks that appeared at 0.99 and 0.73 V during the negative scan were assigned to the reduction of the cations of spirobifluorene and dimeric carbazole, respectively. Consequently, we employed a potential range between −0.8 and 1.0 V for the film deposition; this positive potential can only oxidise the carbazole units, whereas the sufficiently low negative potential ensures the complete reduction of the dimeric carbazyl cations and removal of their counterions (PF_6_^−^) from the films^47^,^48^,^49^. From the second cycle in the continuous CV scan (Figure S1b), the current attributed to the oxidation of dimeric carbazoles at 0.83 V increased with increasing cycle number; thus, more dimeric carbazole formed and the POP films progressively grew on the electrode^43^,^44^,^45^,^46^.

The electro-oxidative coupling reaction took place with near 100% efficiency and the resulting POP films were highly cross-linked and insoluble in organic solvents. Infrared, electronic absorption and X-ray photoelectron spectroscopic (XPS) measurements revealed that the content of the monomeric carbazole unit in the films is negligible (Figure S2, S3a and S4). The size and shape of the POP films were determined by the electrodes employed; films of 0.5 × 1.5 cm^2^ were utilised in this study. This method allows good control over the film thickness by simply tuning the number of scan cycles. A series of thin films with controlled thickness were synthesised, where each cycle resulted in an increase in the film thickness of approximately 2 nm (Figure S5 and S6). We conducted Kr adsorption isotherm measurements and observed that the POP films were highly porous and exhibited exceptional Brunauer–Emmett–Teller (BET) surface area of 2190 m^2^ g^−1^ (Figure S7a). The BET surface areas of the POP films were independent of the film thickness (Figure S7b). High-resolution transmission electron microscopy (HR-TEM) revealed that the films had homogeneous microporous textures with pore diameters of approximately 2 nm (Figure S8). Our strategy offers a high throughput method of synthesising highly cross-linked POP films with controlled thickness.

### Electronic features of POP films

The as-synthesized POP films retained the absorption band of the spirobifluorene unit at 350 nm and also displayed an absorption band at 310 nm, assignable to the dimeric carbazole, which was red shifted by 22 nm from the band of the monomeric carbazole unit of TPSC (Figure S3a). This redshift, together with an enhanced absorption coefficient, indicated extended π-conjugation in the electronic structure of the films. Upon excitation at 350 nm, the films emitted a brilliant blue luminescence at 434 nm (Figure S3b), with an absolute fluorescence quantum yield of 19%. The POP films exhibited the same absorption and emission spectra and had similar fluorescence quantum yields, irrespective of film thickness. We observed that the films significantly depolarised the fluorescence, resulting in a polarisation value of only 0.012, whereas TPSC exhibited a much larger polarisation value of 0.052 (Figure S3c). These observations suggest that the photogenerated excitons in the POP films are not localised but can migrate over the three-dimensional π-network^19^. Exciton migration has been observed to facilitate the transfer of energy in both natural and artificial light-harvesting systems^50^.

### Light harvesting and one-step energy transduction

**Figure 2:**
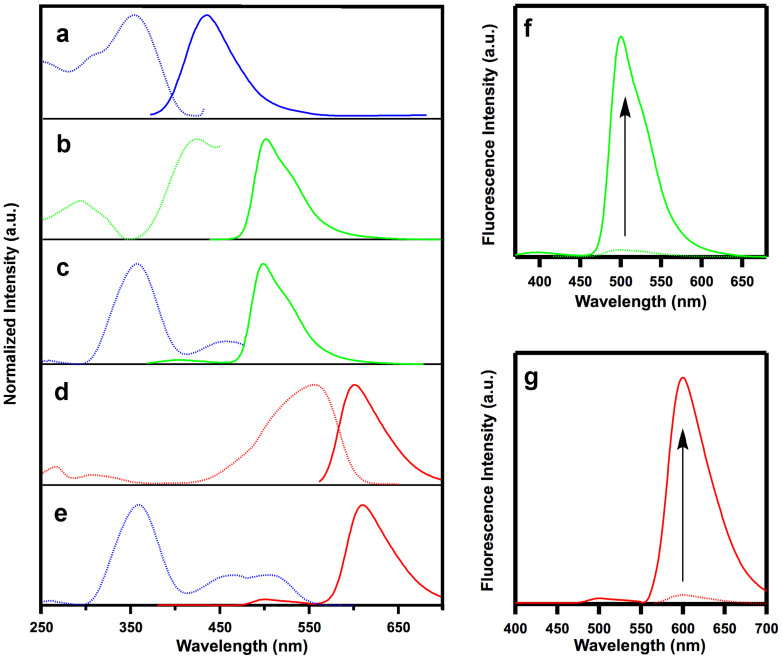
Fluorescence excitation and emission spectra. (a–e), Normalized fluorescence excitation (dotted curve) and emission (solid curve) spectra of (a) the POP films (dotted blue curve monitored at 434 nm, solid blue curve upon excitation at 350 nm), (b) coumarin 6 (dotted green curve monitored at 500 nm, solid green curve upon excitation at 423 nm, in CH_2_Cl_2_), (c) the POP⊃C6 films (3.19 mol% coumarin 6; dotted green curve monitored at 500 nm, solid green curve upon excitation at 350 nm), (d) Nile red (dotted red curve monitored at 600 nm, solid red curve upon excitation at 556 nm, in CH_2_Cl_2_), and (e) the POP⊃C6×NR films (3.19 mol% coumarin 6 and 5.41 mol% Nile red; dotted red curve monitored at 608 nm, solid red curve upon excitation at 360 nm). (f), Amplified fluorescence intensity of the POP⊃C6 film (3.19 mol% coumarin 6) upon excitation of the POP framework (green curve) and coumarin 6 itself (dotted green curve). (g), Amplified fluorescence intensity of the POP⊃C6×NR film (3.19 mol% coumarin 6 and 5.41 mol% Nile red), upon excitation of the POP framework (red curve) and Nile red itself (dotted red curve).

The micropores of the POP films are useful for docking laser dye molecules that enable the POP skeletons to harvest photons and channel the excitation energy to the dye molecules with high efficiency, thus building up one-step energy transduction systems. To select a suitable candidate for the laser dye, the paring of the excitation and emission bands was investigated using fluorescence spectroscopy (Figure 2). The excitation and emission wavelengths of the POP films were 350 and 434 nm, respectively (Figure 2a), whereas those of the green laser dye coumarin 6 (C6) were centered at 421 and 500 nm, respectively (Figure 2b). Therefore, the emission band of the POP films is overlapped with the excitation band of coumarin 6 to a large degree, thereby allowing the formation of a singlet-to-singlet energy transfer pathway from the POP antennae to the coumarin 6 energy acceptor^50^. We prepared the POP⊃C6 films by immersing the POP films into CH_2_Cl_2_ solutions containing different concentrations of coumarin 6 for 12 h and subsequently rinsed them with CH_2_Cl_2_ until the rinse solution was still clear after use. We optimised the film thickness for energy transfer and observed that 20-nm thick films could be tuned to have various coumarin 6 contents and that the energy transfer process could be managed in a controlled manner (Figure S9). Thus, twelve 20-nm thick films were prepared to bear different loadings ranging from 0 to 3.19 mol% of coumarin 6, as determined by XPS measurements (Figure S10a, b and Table S1). However, the maximum coumarin 6 content that could be attained was 15.1 mol% (Figure S10i, j), by virtue of the high surface area of the POP films.

**Figure 3:**
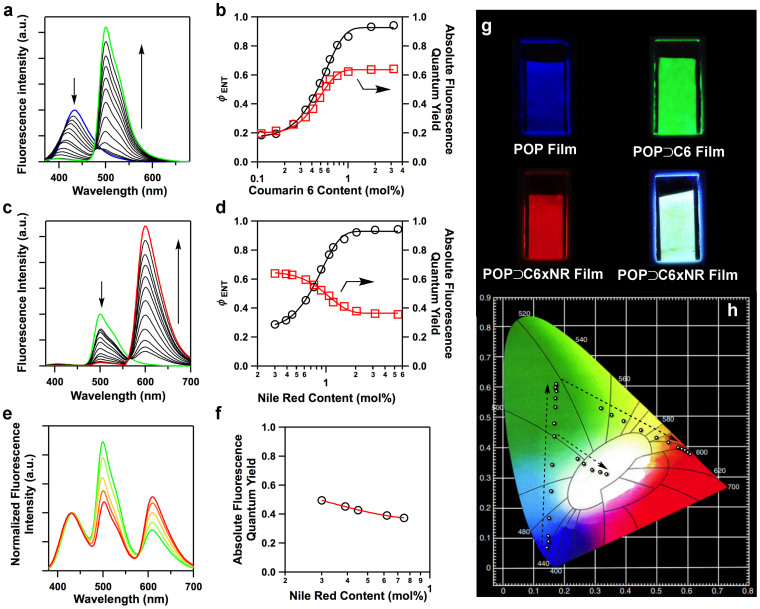
Cascade energy transfer. (a), One-step energy transfer from POP network to coumarin 6 as evident by the fluorescence spectral changes of the POP film (20-nm thick) containing varied coumarin 6 content (0 to 3.19 mol%) upon excitation of the POP skeleton. (b), Energy-transfer efficiencies from the POP film to coumarin 6 (left *y*-axis) and the absolute fluorescence quantum yields of the POP⊃C6 films (right *y*-axis) are controlled by the content of coumarin 6. (c), Cascade energy transfer engines with complete energy transfer as evident by the fluorescence spectral changes of the POP⊃C6×NR films (3.19 mol% coumarin 6) with varying Nile red content (0 to 5.41 mol%) upon excitation of the POP skeleton. (d), Total energy-transfer efficiencies from the POP⊃C6 films to coumarin 6 and Nile red (left *y*-axis) and absolute fluorescence quantum yields of the POP⊃C6×NR films(right *y*-axis) are controlled by the content of Nile red. (e), Cascade energy transfer engines with incomplete energy transfer as evident by the normalized fluorescence spectra of the POP⊃C6×NR films (0.63 mol% coumarin 6) with varying Nile red content (0.30 to 0.75 mol%) upon excitation of the POP skeleton. (f), Absolute fluorescence quantum yields of the white luminescent POP⊃C6×NR films (right *y*-axis) are controlled by the relative dye contents. (g), Photos of the films exhibiting three primary emission colours and white luminescence under a UV lamp. (h), Luminescence engineering of thirty nanofilms traced by the CIE coordinates (dots on the map; arrows represent the trace).

The energy transduction process was monitored by fluorescence spectroscopy. As the coumarin 6 content was increased from 0 to 3.19 mol%, the green luminescence of coumarin 6 increased in intensity, whereas the blue emission from the POP skeletons decreased, giving rise to directed energy funneling from POP network to coumarin 6 and producing a series of luminescence colours gradually engineered to range from deep blue to brilliant green (Figure 3a). Usually coumarin 6 in solid or aggregated states exhibits different luminescence behaviour from that in dilute solution (monomeric state) by showing characteristic redshifted emission band and decreased luminescent efficiency. Notably, the emission wavelength at 500 nm observed for the POP⊃C6 films is the same as that of monomeric coumarin 6, which indicates that the coumarin 6 molecules are segregated from each other and “independently” confined in the pores. These luminescence spectral changes revealed the energy transfer from the POP skeletons to coumarin 6. Indeed, the efficiency (*Φ*_ENT_) increased in a sigmoidal fashion and reached 94% as the coumarin 6 content approached 3.19 mol% (Figure 3b), while the energy transfer speed is solely dependent on the coumarin 6 content (see below). For the POP⊃C6 film (3.19 mol% coumarin 6), approximately 30 TPSC units (calculated by 94/3.19) harvested the photons and funnelled the excitation energy to one coumarin 6 molecule, demonstrating an outstanding antenna effect. As an example, the green luminescence intensity of the POP⊃C6 film (3.19 mol% coumarin 6) upon excitation of the POP skeletons is 31-fold higher than that measured from the direct excitation of coumarin 6 itself (Figure 2f). As a control, the spin-coated TPSC films did not exhibit good energy transfer (Figure S11). Because coumarin 6 exhibits an absolute fluorescence quantum yield (76%) that is higher than that of the POP film (19%), the enhanced energy transfer efficiency of the dosed films, taking the site isolation effect into account, effectively increases the fluorescence quantum yields of the POP⊃C6 films (Figure 3b). The green-luminescent POP⊃C6 film (3.19 mol% coumarin 6; Figure 3g) exhibited a fluorescence quantum yield as high as 64%.

### Light harvesting and cascade energy-transfer engine with complete energy transfer

The controlled one-step energy transfer and remarkable antenna effect promoted us to explore the possibility of constructing cascade energy transfer engine. To build up such an engine, we introduced a second laser dye as energy-accepting counterpart to constitute a two-step energy transfer and relay system. We observed that the laser dye Nile red (NR) serves as an energy-accepting counterpart when trapped within the pores of the POP⊃C6 films. Nile red emits a deep-red fluorescence at 600 nm with an excitation band at 554 nm (Figure 2d); this excitation wavelength overlaps with the emission of the POP⊃C6 films and thus could be used to form a cascade energy transfer from the POP network to coumarin 6 and then relay from coumarin 6 to Nile red. We prepared twelve POP⊃C6×NR films for luminescence engineering by fixing the coumarin 6 content at 3.19 mol% and tuning the Nile red loading from 0 to 5.41 mol% (Figure S10c-f and Table S1). The XPS spectra of the POP⊃C6×NR films demonstrated that no coumarin 6 is released into the solvent during the loading of Nile red (Figure S10d, f and Table S1). This result is reasonable because the film adopts a 3D crosslinked network structure, which prevents the leach of the dyes once loaded. The film is highly porous, in which only few pores trap coumarin 6, as indicated by the content of dyes in the films. Therefore, the presence of coumarin 6 trapped only in a few pores does not affect the diffusion of Nile red into the films; there are still many pores that are open and accessible to Nile red in the films. Notably, a maximum Nile red loading of 19.4 mol% was attained (Figure S10k, l and Table S1).

The cascade energy transfer process is easily evident by the colour change in luminescence. Upon excitation of the POP skeleton, the POP⊃C6×NR films exhibited increased red emission from Nile red and decreased green emission from coumarin 6 as the content of Nile red increased from 0 to 5.41 mol%, giving rise to tuned emission spanning from bright green to deep red (Figures 3c and 3g). The energy transfer from the POP framework to coumarin 6 is almost complete (*Φ*_ENT_ = 94%), whereas the energy relay from coumarin 6 to Nile red is solely dependent on the content of Nile red with an increased efficiency in a sigmoidal fashion reaching 93% as the Nile red content increased to 5.41 mol% (Figure 3c). Remarkably, the high *Φ*_ENT_ values for the cascade energy transfer gave rise to a significant antenna effect; the POP⊃C6×NR film emitted a 24-fold amplified red luminescence upon excitation of the POP skeleton in comparison with the direct excitation of Nile red (Figure 2g). The fluorescence quantum yields of the green-to-red luminescent films were dependent on the Nile red content and changed from 64% for green luminescence to 36% for red luminescence (Figure 3c), which was very close to that of monomeric Nile red (38%). The similarities in the fluorescence wavelengths and quantum yields of the POP⊃C6×NR films and monomeric Nile red moieties indicate that the Nile red molecules are spatially segregated from other dye molecules and are “independently” confined within the nanopores. Therefore, the POP films can dock two types of laser dye molecules in tailored combinations and in a segregated manner, resulting in complete energy transfer and manageable energy relay systems that enable a new mechanism for blue-to-green-to-red luminescence engineering.

### Light harvesting and cascade energy-transfer engine with incomplete energy relay

The above POP films enable complete energy transfer from POP network to coumarin 6 and tunable energy relay from coumarin 6 to Nile red. The cascade engines were further explored to manipulate the efficiency and speed (see below) of two steps by reducing the energy transfer efficiency. The incomplete energy transfer would leave blue, green and red luminescence, opening a window to produce white emission. For this purpose, we decreased and optimised the coumarin 6 content at 0.63 mol%, whereas the Nile red content was tuned from 0.3 mol% to 0.75 mol% (Figure S10g, h, Table S1). The energy-transfer efficiency from the POP film to coumarin 6 is 71% and the relay efficiency from coumarin 6 to Nile red is 29–55% (Figure 3e). The measured absolute fluorescence quantum yields of these five white emissions ranged from 37% to 50% (Figure 3f). Remarkably, the POP⊃C6×NR film containing 0.75 mol% Nile red emitted a white light (Figure 3g) with Commission Internationale de L'Eclairage (CIE) coordinates of (0.33, 0.31) (Figure 3h), which is very close to the CIE coordinates (0.33, 0.33) of standard pure white light.

### Luminescence engineering traits

We plotted the luminescent colour tracks of the aforementioned thirty POP films by mapping their CIE coordinates (Figure 3h). The original POP film has CIE coordinates of (0.15, 0.06), which change along a straight line (Figure 3h, white arrow) between the original POP film and coumarin 6 (0.18, 0.60), as the coumarin 6 content increases. Therefore, twelve different luminescence colours, including blue, sky blue, blue green and green, were produced upon docking with various amounts of coumarin 6. By further docking Nile red in the films, the CIE coordinates changed along another line (Figure 3h, white arrow) between coumarin 6 (0.18, 0.60) and Nile red (0.62, 0.38). Along this line, we demonstrated that twelve different luminescence colours, including green, yellow-green, yellow, orange, red and deep-red, were successfully engineered. In addition, the POP⊃C6×NR films can be engineered with coumarin 6 at content levels other than the loading of 3.19 mol% used to make the range of previously described films, which would allow the predesigned generation of various luminescent colours. As exemplified by the POP⊃C6×NR film containing 0.63 mol% coumarin 6, white luminescence can also be engineered along a new track on the CIE map (Figure 3h, black arrow).

### Mechanistic insights

We calculated the effective distance between coumarin 6 and Nile red in the cascade energy relay process. We assumed that four TPSC molecules circled a pore (in agreement with the simulation and BET results), leading to a pore size of 2.3 nm. For the POP⊃C6×NR film with 3.19 mol% coumarin 6 and 5.41 mol% Nile red, the maximum and minimum distances between coumarin 6 and Nile red molecules were estimated to be approximately 9 and 4 nm, respectively. The distance between coumarin 6 and Nile red is much lower than the Förster distance of energy transfer, thus enabling the cascade energy relay to occur effectively within the POP⊃C6 × NR films.

**Figure 4:**
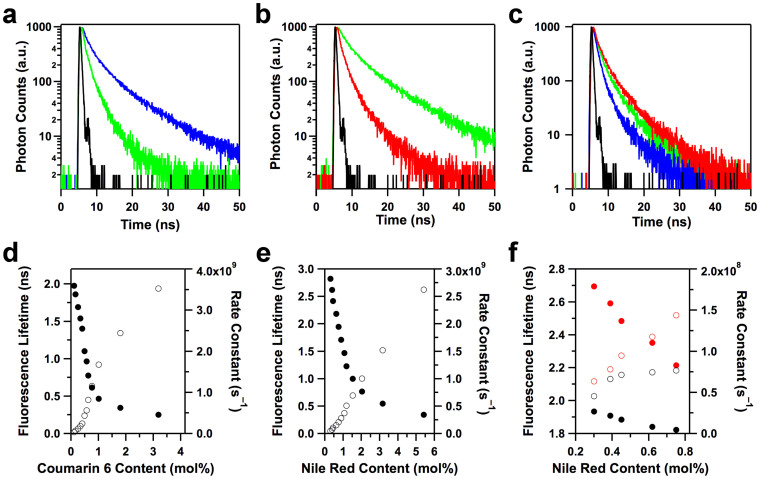
Control over the cascade energy-transfer speed. (a), Fluorescence decay curves of the POP film (blue curve) and POP⊃C6 film (3.19 mol% coumarin 6, green curve) monitored at 410 nm. (b), Fluorescence decay profiles of the POP⊃C6 film (3.19 mol% coumarin 6, green curve) and cascade energy transfer POP⊃C6×NR film (3.19 mol% coumarin 6 and 5.41 mol% Nile red, red curve) monitored at 503 nm. (c), Fluorescence decay curves of the cascade energy transfer and white-luminescent POP⊃C6×NR films (0.63 mol% coumarin 6 and 0.75 mol% Nile red) monitored at 430 (blue curve), 503 (green curve) and 600 nm (red curve). Black curves in (a–c) are instrument response function profiles. (d), Fluorescence lifetime (filled circles) and rate constant of energy transfer (open circles) of the one-step energy transfer POP⊃C6 films. (e), Fluorescence lifetime (filled circles) and rate constant of energy transfer (open circles) of the cascade energy transfer POP⊃C6×NR films with complete energy transfer. (f), Fluorescence lifetime (filled circles) and rate constant of energy transfer (open circles) monitored at 410 (black) and 503 nm (red) of the cascade energy transfer and white-luminescent POP⊃C6×NR films with incomplete energy transfer.

To gain insight into the energy transfer and cascade energy relay dynamics, we conducted time-resolved fluorescence spectroscopy of the POP, POP⊃C6 and POP⊃C6×NR films upon excitation of the POP skeletons. The POP films exhibited an average lifetime (*τ*_0_) of 2.12 ns (Figure 4a, blue curve). As the coumarin 6 content was increased from 0.11 mol% to 3.19 mol% (Figure 4a, green curve), the lifetime (*τ*_DA_) of the POP⊃C6 films monitored at 410 nm of the POP emission spectrum, was significantly decreased from 1.98 to 0.25 ns in a sigmoidal fashion (Figure 4d, solid circles; Table 1). Interestingly, the rate constant of energy transfer (*k*_ENT_)^50^ from the POP skeletons to coumarin 6, as calculated using the equation *k*_ENT_ *= τ*_DA_^−1^ − *τ*_0_^−1^, increased from 0.03 × 10^9^ to 3.5 × 10^9^ s^−1^ (Table 1). Figure 4d (open circles) clearly shows that the *k*_ENT_ values are controlled by the content of coumarin 6 in the POP films. Therefore, in the one-step energy transfer nanofilms, both the efficiency and the speed are solely determined by the coumarin 6 content.

In the cascade energy transfer nanofilms, the energy transfer efficiency and speed were controlled by the relative contents of coumarin 6 and Nile red. In the case of the complete energy transfer films (3.19 mol% coumarin 6), the lifetime (*τ*_0_) monitored at 503 nm on the coumarin 6 emission spectrum was 3.25 ns (Figure 4b, green curve). As the Nile red content increased from 0.30 mol% to 5.41 mol% (Figure 4b, red curve), the POP⊃C6×NR films exhibited a considerably decreased lifetime (*τ*_DA_), and finally reached a value of only 0.34 ns (Figure 4e, solid circles; Table 1). The values of *k*_ENT_ from coumarin 6 to Nile red drastically increased from 0.05 × 10^9^ to 2.6 × 10^9^ s^−1^ (Figure 4e, open circles; Table 1). The POP⊃C6×NR films consist of a two-step cascade energy transfer process from the POP film to coumarin 6 and coumarin 6 to Nile red. Because these POP⊃C6×NR films have the high coumarin 6 content (3.19 mol%) and allow for complete energy transfer from the POP film to coumarin 6 with a *k*_ENT_ value of 3.5 × 10^9^ s^−1^, the rate-determining step in the cascade energy-transfer process is the energy-relay step from coumarin 6 to Nile red.

For the white luminescent films (0.63 mol% coumarin 6, 0.30 to 0.75 mol% Nile red), the energy transfers from the POP film to coumarin 6 and relay from coumarin 6 to Nile red are both incomplete processes (Table 1). The film made with 0.75 mol% Nile red exhibited three different decay profiles when monitored at 410, 503 and 600 nm, whereas their lifetimes were 1.82, 2.21 and 3.14 ns, respectively (Figure 4c). The average lifetime of the POP films (blue emission) was decreased from 1.93 to 1.82 ns (Figure 4f, filled black circles), whereas the lifetime of coumarin 6 was decreased from 2.69 to 2.21 ns (Figure 4f, open black circles). Therefore, the rate constant for the energy transfer from the POP film to coumarin 6 was increased from 4.5 × 10^7^ to 7.7 × 10^7^ s^−1^ (Figure 4f, filled red circles), whereas the rate constant for the energy transfer from coumarin 6 to Nile red was increased from 6.4 × 10^7^ to 1.44 × 10^8^ s^−1^ (Figure 4f, open red circles). In the white luminescence films, the cascade energy-transfer process is also controlled in terms of both rate constant and efficiency by tuning the dye content of the film, and energy transduction from the POP film to coumarin 6 becomes the rate-determining step.

The cascade energy-transfer processes are controlled over a range of efficiencies and a rather wide range of rate constants – from 10^7^ to 10^9^ s^−1^ – by tuning the nature and content of the dye incorporated into the film. Remarkably, the rate-determining step of cascade energy transfer is solely dependent on the relative amounts of dyes in the films. Our results reveal that the films trigger vectorial and highly efficient cascade energy transfer through their channels at a high yet manageable speed, which enables predesignable and precise engineering of their luminescence. Because dye molecules in this system can be replaced with redox-active components, the cascade films can be useful for the development of photocatalytic and photoenergy conversion systems.

## Conclusions

We have integrated controlled cascade energy transfer and relay engines into light-harvesting POP films. The porous polymer network with extended π-cloud delocalisation increases the interface for exciton pumping and energy transfer, whereas the nanopores offer ‘independent rooms' for the spatially segregated molecular dockings of laser dyes. The synthetic control over the incorporated dye components and their relative compositions results in the generation of controlled cascade energy transfer systems that can be tuned in a straightforward and precise manner. The efficiency and speed of the cascade energy transfer were controlled by the dye components and their loading in the films and were exploited to achieve energy-transfer efficiencies of near 100% and energy-transfer rate on the order of 10^9^ s^−1^. We demonstrate the accuracy and versatility of luminescence engineering by developing films that luminesce with thirty different emission hues, including the three primary colours and pure white. Another significant finding is that the films gain the highest fluorescence quantum efficiencies of the corresponding laser dyes as a result of site segregation effect, whereas the antenna effect of the POP skeletons greatly amplifies the green and red emissions by 30- and 24-fold compared with the emissions generated by direct excitation of the laser dyes. Our strategy may open new possibilities for the development of cascade systems for luminescent and lasing materials, artificial photosynthesis and photoenergy conversion.

## Methods

### POP films

The electro-oxidative coupling reaction was performed using a standard one-compartment, three-electrode electrochemical cell attached to an ALS/CHi 610C electrochemical workstation. The Ag/Ag^+^ nonaqueous electrode was used as the reference electrode. ITO was used as the working electrode, and titanium metal was used as the counter electrode. A mixture of TPSC (0.5 mg mL^−1^) and TBAPF_6_ (0.1 M) in CH_2_Cl_2_ (5 mL) was used as the electrolyte solution. The CV potential range was set between −0.8 and 1.0 V, and the scan rate was 50 mV s^−1^. The thickness of the films was controlled by the cycle number used in their formation. The resulting films were washed with acetonitrile to remove unreacted precursors and supporting electrolytes, dried under vacuum and stored under Ar in the absence of light.

### POP⊃C6 films

The POP⊃C6 films were prepared by immersing the POP films into CH_2_Cl_2_ solutions (20 mL) of coumarin 6 at different concentrations (1 × 10^−5^, 2 × 10^−5^, 3 × 10^−5^, 4 × 10^−5^, 5 × 10^−5^, 6 × 10^−5^, 7 × 10^−5^, 8 × 10^−5^, 9 × 10^−5^, 1 × 10^−4^, 2 × 10^−4^ and 3 × 10^−4^ mol L^−1^) for 12 h at room temperature. The resulting films were rinsed several times with CH_2_Cl_2_ until the wash solution was still clear after use. The resulting films were dried under vacuum and stored under Ar in the absence of light. The loading of coumarin 6 in the POP⊃C6 films were determined by XPS mesurements are 0.11 mol%, 0.16 mol%, 0.25 mol%, 0.34 mol%, 0.41 mol%, 0.49 mol%, 0.57 mol%, 0.57 mol%, 0.63 mol%, 0.76 mol%, 1.01 mol%, 1.80 mol% and 3.19 mol% (Table S1).

### POP⊃NR films

The POP⊃NR films were prepared by immersing the POP films into CH_2_Cl_2_ solutions (20 mL) of Nile red at different concentrations (1 × 10^−5^, 2 × 10^−5^, 3 × 10^−5^, 4 × 10^−5^, 5 × 10^−5^, 6 × 10^−5^, 7 × 10^−5^, 8 × 10^−5^, 9 × 10^−5^, 1 × 10^−4^, 2 × 10^−4 ^and 3 × 10^−4^ mol L^−1^) for 12 h at room temperature. The resulting POP⊃C6 films were rinsed several times with CH_2_Cl_2_ until the wash solution was still clear after use, at which point the films were dried under vacuum and stored under Ar in the absence of light. The loadings of Nile red in the POP⊃NR films were determined by XPS mesurements; the results were summarized in Table S1. These POP⊃NR films were utilised as the standard to determine the XPS signals of Nile red in the POP⊃C6×NR films (Table S1).

### POP⊃C6×NR films

The POP⊃C6×NR films (coumarin 6 content = 3.19 mol%) for green-to-red luminescence engineering were prepared by immersing the POP⊃C6 films into CH_2_Cl_2_ solutions (20 mL) of Nile red at different concentrations (1 × 10^−5^, 2 × 10^−5^, 3 × 10^−5^, 4 × 10^−5^, 5 × 10^−5^, 6 × 10^−5^, 7 × 10^−5^, 8 × 10^−5^, 9 × 10^−5^, 1 × 10^−4^, 2 × 10^−4 ^and 3 × 10^−4^ mol L^−1^). The POP⊃C6×NR films (coumarin 6 content = 0.63 mol%) used in engineering the luminescence of white light were prepared by immersing the POP⊃C6 films into CH_2_Cl_2_ solutions (20 mL) of Nile red at different concentrations (1 × 10^−5^, 2 × 10^−5^, 3 × 10^−5^ and 4 × 10^−5^ mol L^−1^) for 12 h at room temperature. The resulting POP⊃C6×NR films were rinsed with CH_2_Cl_2_ until the wash solution was still clear after use, dried under vacuum and stored under Ar in the absence of light. For the POP⊃C6×NR films (coumarin 6 content = 3.19 mol%) used in green-to-red luminescence engineering, the Nile red content was 0.3 mol%, 0.39 mol%, 0.45 mol%, 0.62 mol%, 0.75 mol%, 0.89 mol%, 1.07 mol%, 1.18 mol%, 1.53 mol%, 2.04 mol%, 3.16 mol% and 5.41 mol%, respectively (Table S1). For the POP⊃C6×NR films (coumarin 6 content = 0.63 mol%) for white luminescence engineering, the Nile red content was 0.30 mol%, 0.39 mol%, 0.45 mol%, 0.62 mol% and 0.75 mol%, respectively (Table S1).

## Supplementary Material

Supplementary Information Supplementary Information

## Figures and Tables

**Table 1:** Fluorescence lifetime and rate constant of energy transfer

One-step energy transfer POP⊃C6 films													
**C6 content**^a^	0	0.11	0.16	0.25	0.34	0.41	0.49	0.57	0.63	0.76	1.01	1.8	3.19
***τ*(ns)**	2.12	1.98	1.86	1.69	1.54	1.40	1.10	0.96	0.78	0.61	0.46	0.34	0.25
***k*_ENT_ (10**^**9**^** s^−1^)**	–	0.03	0.06	0.12	0.18	0.24	0.44	0.56	0.82	1.16	1.68	2.44	3.53

Cascade energy transfer POP⊃C6×NR films with complete energy transfer													
**NR content**^a^	0	0.30	0.39	0.45	0.62	0.75	0.89	1.07	1.18	1.53	2.04	3.16	5.41
***τ*(ns)**	3.25	2.82	2.62	2.41	2.18	1.94	1.71	1.47	1.22	1.00	0.76	0.55	0.34
***k*_ENT_ (10**^**9**^** s^−1^)**	–	0.05	0.07	0.11	0.15	0.21	0.28	0.37	0.51	0.69	1.00	1.52	2.62

ain mol%.
