# Supplementary material for: Cascade exciton-pumping engines with manipulated speed and efficiency in light-harvesting porous π-network films
Source: Sci Rep. 2015 Mar 9;5:8867. doi: 10.1038/srep08867 (PMC5390074; doi:10.1038/srep08867)
Supplement: Supplementary Information [file srep08867-s1.pdf]

Supplementary Information

Cascade exciton-pumping engines with manipulated speed and efficiency in light-harvesting porous π-network films

Cheng Gu,[†] Ning Huang,[†] Fei Xu, Jia Gao and Donglin Jiang

Department of Materials Molecular Science, Institute for Molecular Science, National Institutes of Natural Sciences, 5-1 Higashiyama, Myodaiji, Okazaki 444-8787, Japan.

[†]These authors contributed equally.

Correspondence and requests for materials should be addressed to D.J. (email: jiang@ims.ac.jp).

Tables of Contents

Supplementary Figures -- *S*2

Supplementary Tables ---*S*12

Supplementary Methods --*S*15

Supplementary References -- *S*19

Supplementary Figures

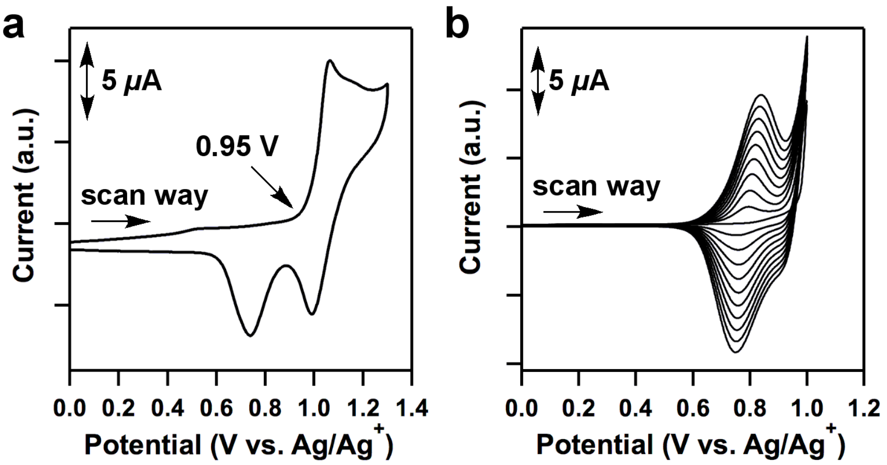

Supplementary Figure S1 | Cyclic voltammetric monitoring of film growth. a and **b**, CV curves of (a) the 1st cycle and (b) 1st to 10th cycle (corresponding to 2-nm t0 20-nm thick films), of a CH_2Cl_2 solution of TPSC in the presence of $TBAPF_6$ electrolyte at 25 °C. The thin films were obtained on ITO electrodes during multicycled CV scans, whereas the film thickness was tuned by controlling the scan numbers.

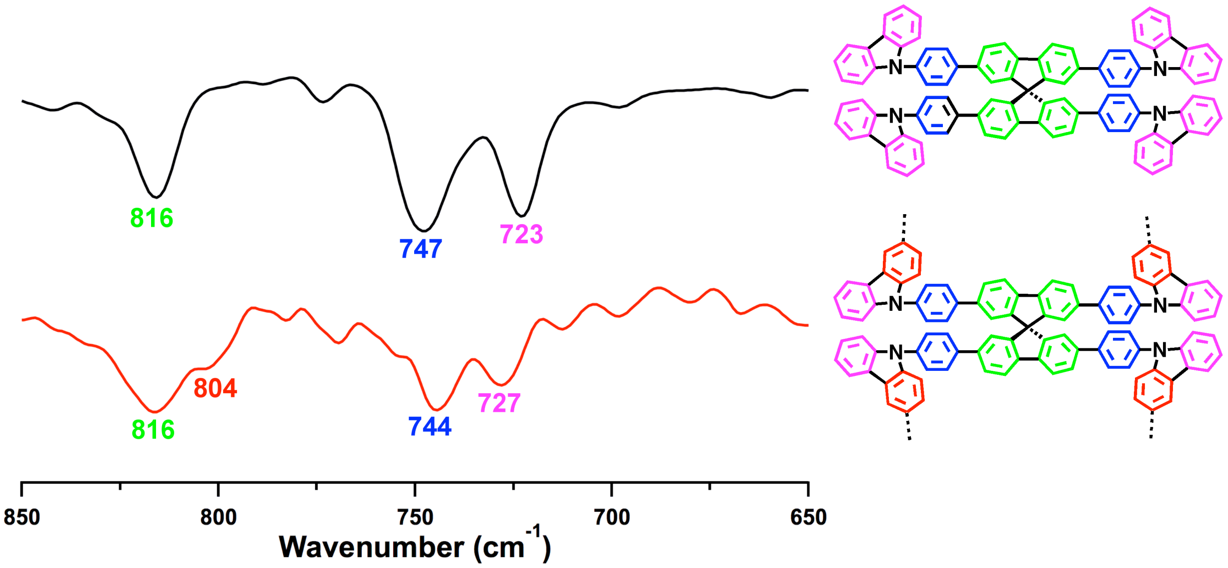

Supplementary Figure S2 | IR spectra. FT IR spectra of the POP film (red curve) and TPSC (black curve). The vibration bands and their corresponding phenyl rings are shown in a same color, and assigned according to reported values[S1,S2].

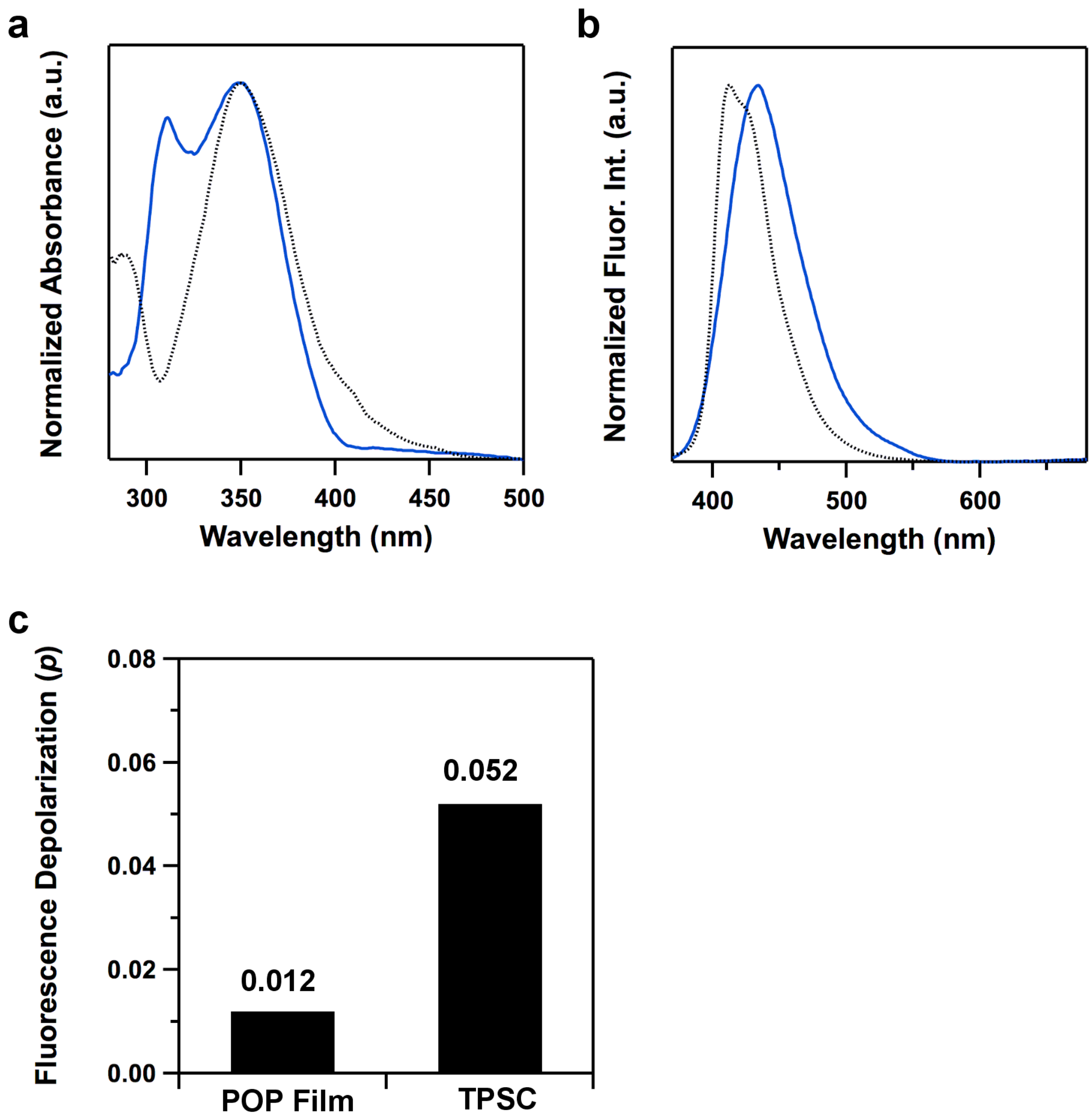

Supplementary Figure S3 | Absorption and fluorescence profiles. (a) UV-vis spectra of the POP films (blue curve) and spin-coated TPSC film (dotted black curve). (b) Fluorescence spectra of the POP film (blue curve) and spin-coated TPSC film (dotted black curve). (c) Fluorescence depolarization (*p*) of the POP film and TPSC.

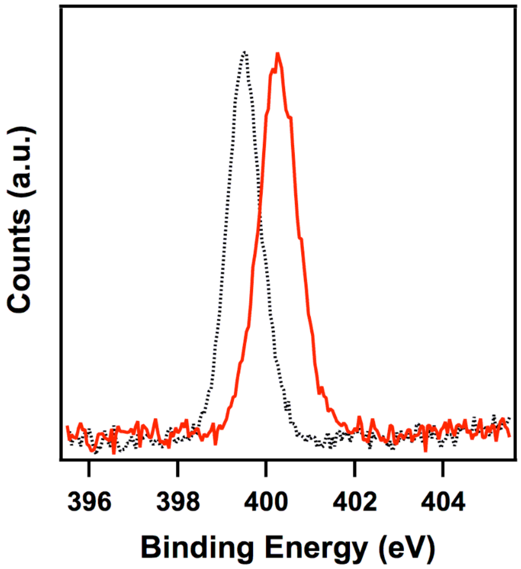

Supplementary Figure S4 | XPS profile. XPS N1s spectra of the POP film (red curve) and spin-coated TPSC film (dotted black curve). The coupling of the carbazole units leads to a shift in the N1s band.

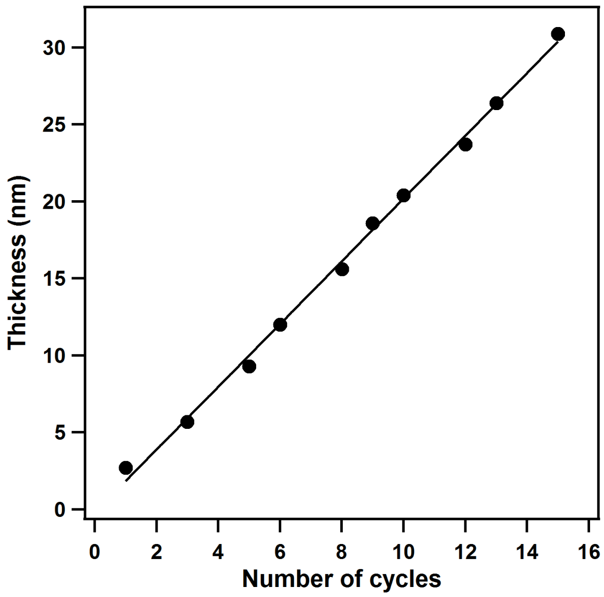

Supplementary Figure S5 | Thickness control. The thickness of the POP films prepared by different CV cycles. The r^2 value for the linear plot is 0.9965.

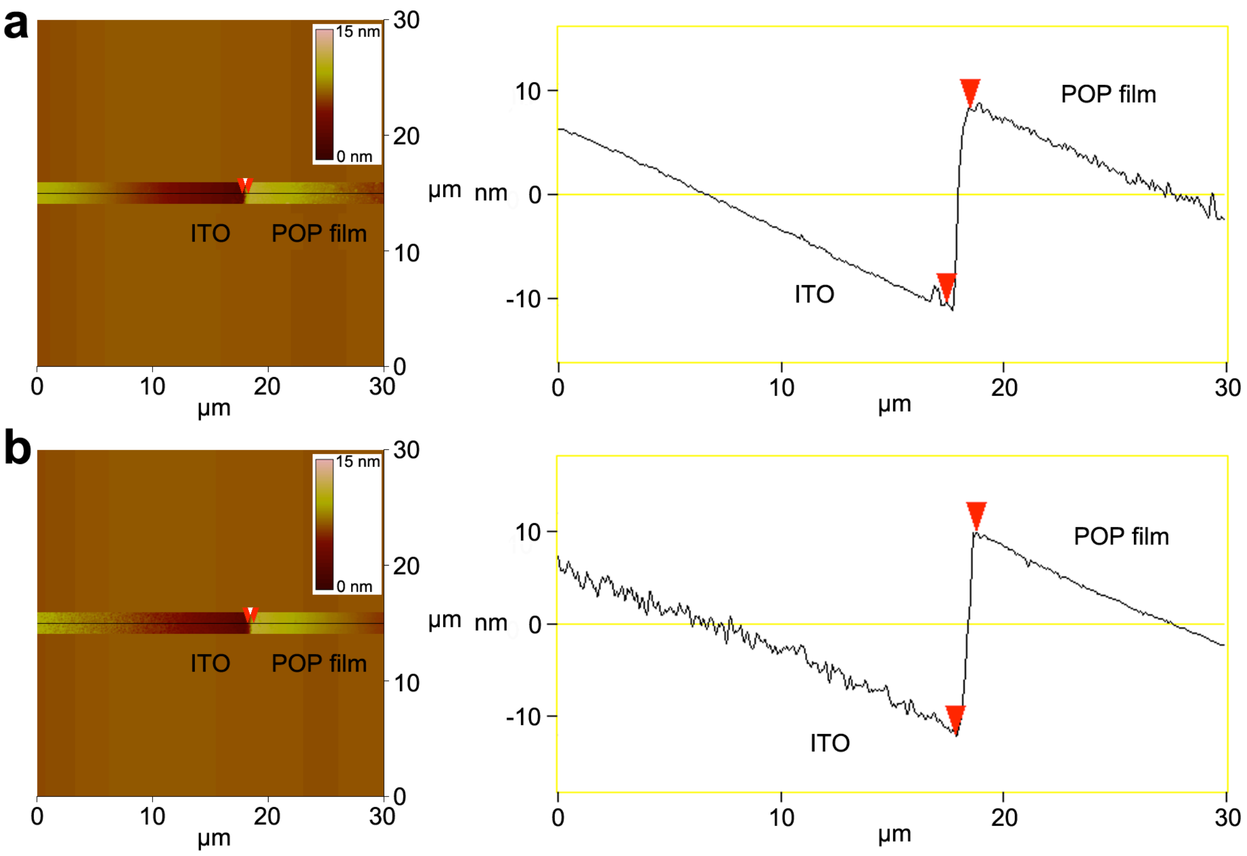

Supplementary Figure S6 | AFM images. The AFM cross-section images of the POP films prepared by (a) 10 and (b) 11 cycles, which have the thickness of 20 and 22 nm, respectively.

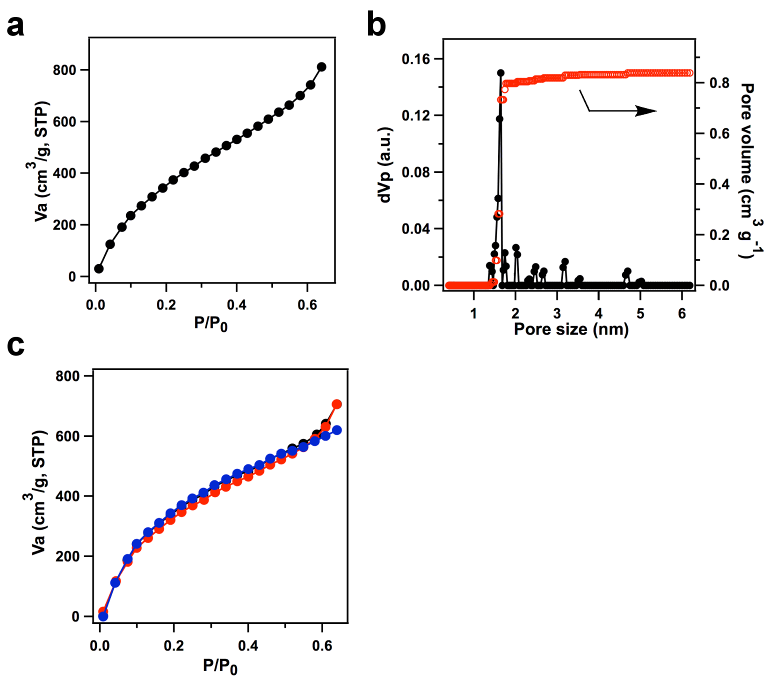

Supplementary Figure S7 | Gas adsorption. (a) Kr sorption isotherm curve of the POP films measured at 77 K. (b) Pore size and pore volume of the POP films. (c) Kr sorption isotherm curves of the POP films with different thickness of 800 nm (black), 1 *u*m (red) and 1.5 μm (blue), which exhibited BET surface area of 2160, 2120 and 2190 m^2 g^{-1}, respectively.

The advantage of Kr over nitrogen gas for the sorption of thin film is widely accepted and clear. Nitrogen at 77 K has a saturation pressure of 760 torr, whereas Kr has a saturation pressure of only 2.5 torr (solid). Since pressure is proportional to the number of molecules in a set volume, there are ~ 300 molecules of nitrogen for every 1 molecule of Kr. When the quantity adsorbed is significantly small, lowering the amount of molecules present by a factor of 300 substantially reduces the amount of error. Considering the above points, the low weight of thin films and the limited space of sample holder, we employed Kr sorption measurements. We added three films on substrates to the sample holder to gain the highest film weight.

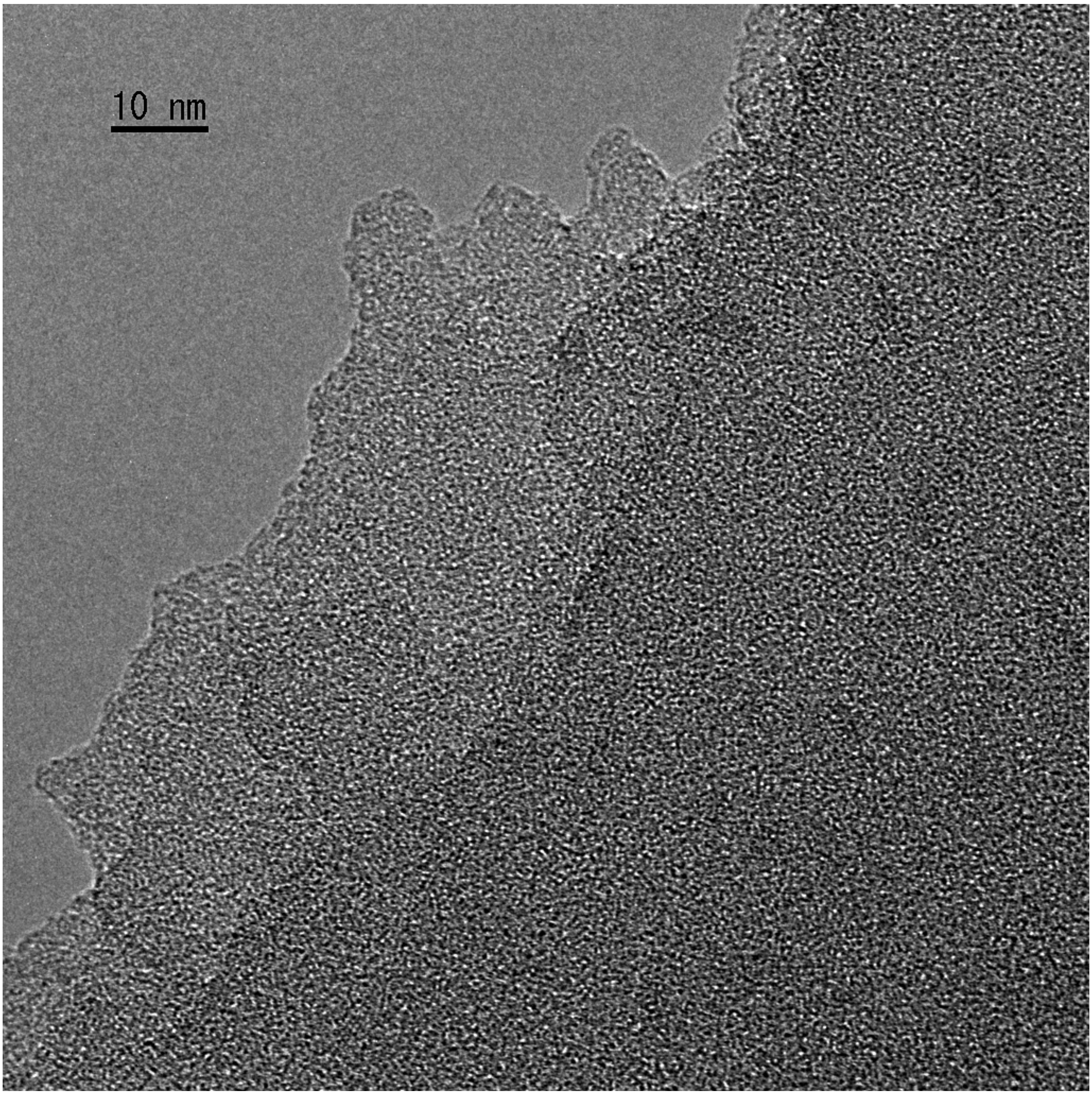

Supplementary Figure S8 | Electron microscopic image. HR-TEM image of the POP film. Microporous texture can be identified.

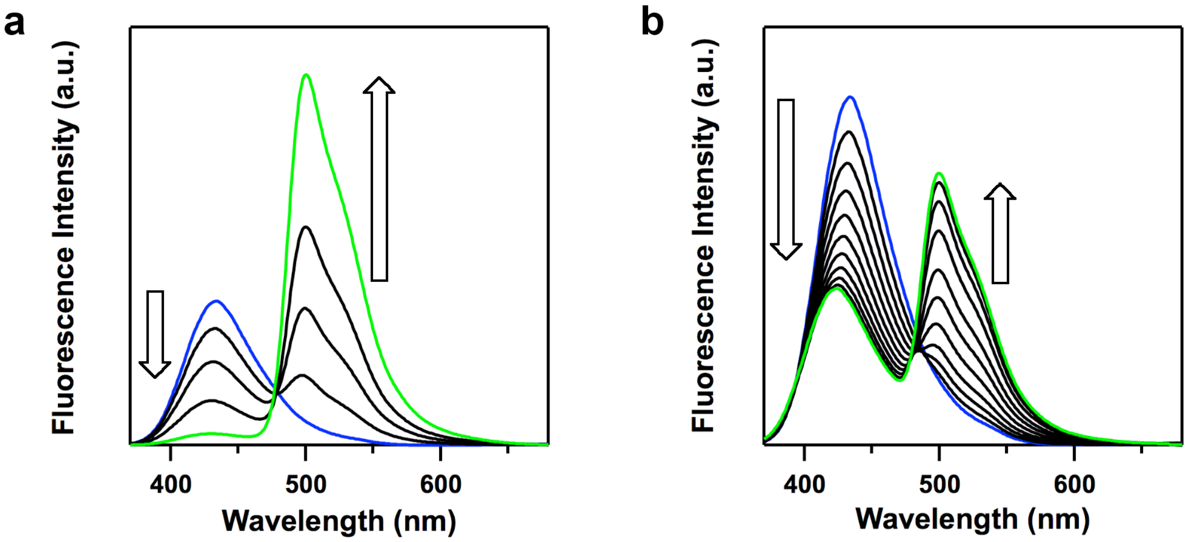

Supplementary Figure S9 | Effect of film thickness on energy transfer. Fluorescence spectral change of the POP⊃C6 films with different coumarin 6 contents in the thickness of (a) 10 nm and (b) 30 nm upon excitation at 350 nm of the POP framework. In the case of 10-nm thick film, even a very low content of coumarin 6 (0.11 mol%, 0.16 mol%, 0.25 mol% and 0.34 mol%) caused a large spectral change, whereas in the case of the 30-nm thick film, the spectral change is quite sluggish and requires a high content of coumarin 6 (0.34 mol%, 0.41 mol%, 0.49 mol%, 0.57 mol%, 0.57 mol%, 0.63 mol%, 0.76 mol%, 1.01 mol%, 1.80 mol% and 3.19 mol%). We thus chose 20-nm thick films for the preparation of the POP⊃C6 (Figure 4a) and POP⊃C6×NR films in this study.

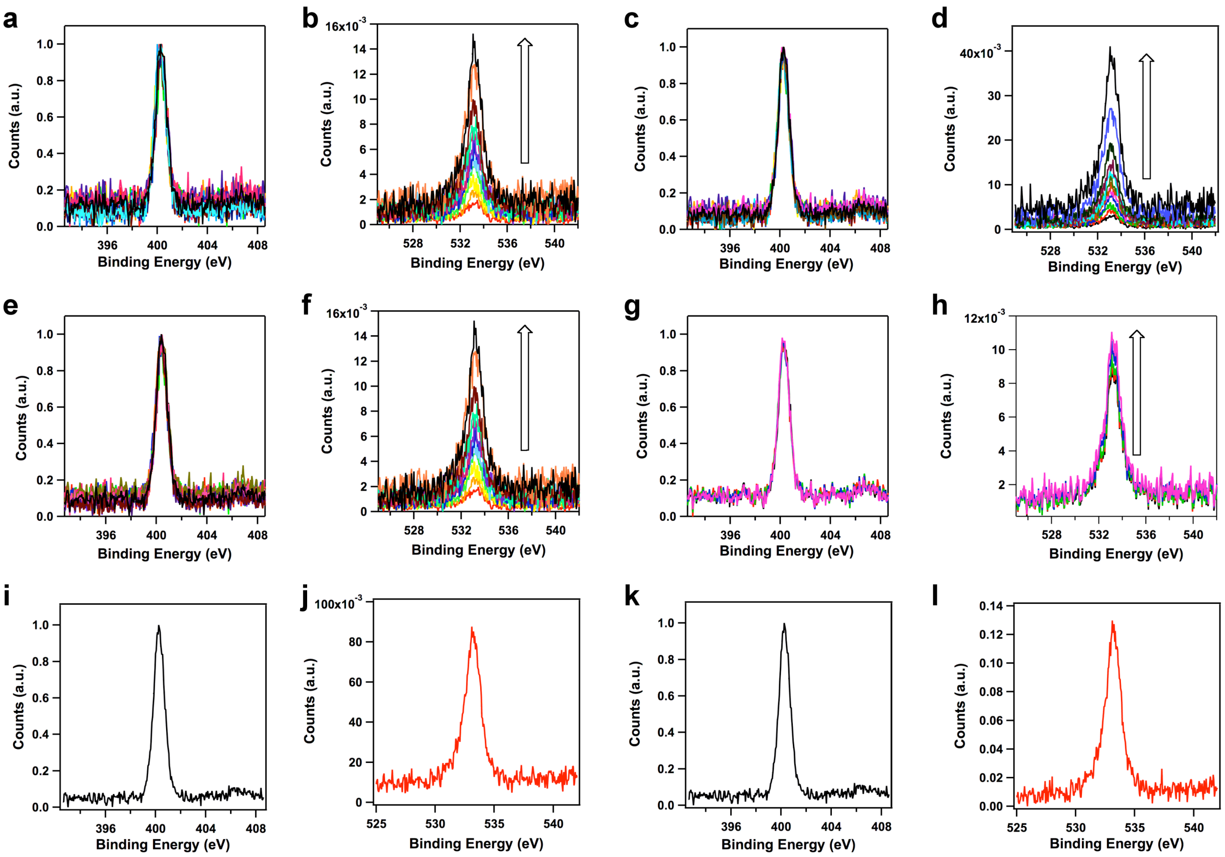

Supplementary Figure S10 | XPS profiles. (a) Normalized N 1s and (b) O 1s XPS spectra of the POP⊃C6 films with different coumarin 6 contents. (c) Normalized N 1s and (d) O 1s XPS spectra of the POP⊃NR films with different nile red contents. (e) Normalized N 1s and (f) O 1s XPS spectra of the POP⊃C6×NR films for green-to-red luminescence engineering. (g) Normalized N 1s and (h) O 1s XPS spectra of the POP⊃C6×NR films for white luminescence engineering. (i) Normalized N 1s and (j) O 1s XPS spectra of the POP⊃C6 film with the maximum coumarin 6 content (15.1 mol%). (k) Normalized N 1s and (l) O 1s XPS spectra of the POP⊃NR film with the maximum nile red content (19.4 mol%).

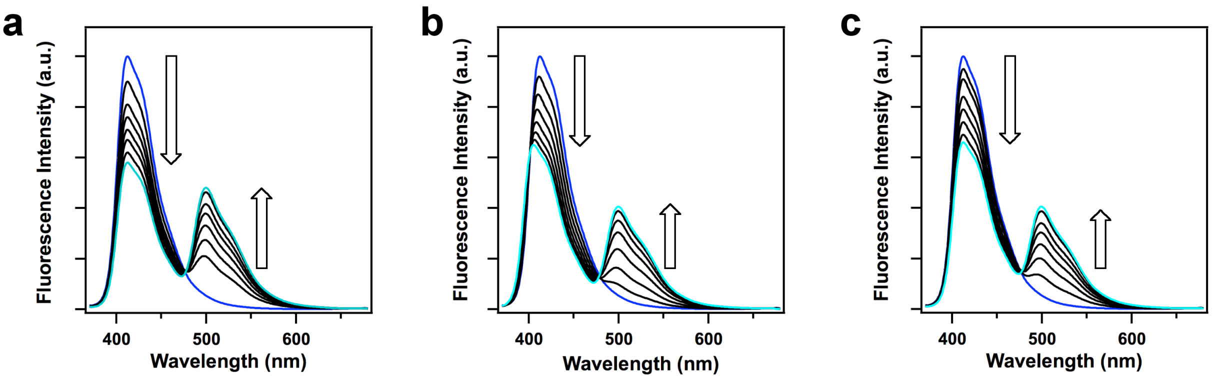

Supplementary Figure S11 | Energy transfer in the spin-coated TPSC films. Fluorescence spectral change of the (a) 10 nm-thick, (b) 20 nm-thick and (c) 30 nm-thick spin-coated TPSC films containing different contents of coumarin 6 (0.49, 0.57, 0.63, 0.76, 1.01, 1.80, and 3.19 mol%), on excitation at 350 nm. The maximum energy transfer efficiency was 58%, 37% and 34% for the 10 nm-, 20 nm- and 30 nm-thick films, respectively.

Supplementary Tables

Supplementary Table S1 | Compositions and components of the POP films

Films	C6 content (mol%)	NR content (mol%)	Films	C6 content (mol%)	NR content (mol%)
The POP⊃C6 films for blue-to-green luminescence engineering	0.11	-	The POP⊃C6 films for blue-to-green luminescence engineering	0.57	-
	0.16	-		0.63	-
	0.25	-		0.76	-
	0.34	-		1.01	-
	0.41	-		1.80	-
	0.49	-		3.19	-
The POP⊃NR films	-	0.30	The POP⊃NR films	-	1.07
	-	0.39		-	1.18
	-	0.45		-	1.53
	-	0.62		-	2.04
	-	0.75		-	3.16
	-	0.89		-	5.41
The POP⊃C6×NR films for green-to-red luminescence engineering	3.19	0.30	The POP⊃C6×NR films for green-to-red luminescence engineering	3.19	1.07
	3.19	0.39		3.19	1.18
	3.19	0.45		3.19	1.53
	3.19	0.62		3.19	2.04
	3.19	0.75		3.19	3.16
	3.19	0.89		3.19	5.41
The POP⊃C6×NR films for white luminescence engineering	0.63	0.30	The maximum C6 content in the POP⊃C6 film: 15.1 mol%	The maximum NR content in the POP⊃NR film: 19.4 mol%	
	0.63	0.39			
	0.63	0.45			
	0.63	0.62			
	0.63	0.75			

The contents of coumarin 6 (C6) and nile red (NR) in the POP films. These films were evaluated by using the XPS measurement for the content of the dyes in the POP films. Coumarin 6 and nile red contains oxygen and nitrogen atoms, whereas the POP film contain nitrogen atom. The contents of coumarin 6 and nile red in the POP films were determined by their ratio of oxygen to nitrogen of the POP films. In detail, the contents were calculated by using the equation of $C_X = (I_X/SF_X)/(\Sigma\ I_X/SF_X)$, where I_X is the normalized area of the XPS bands for the measured element X, and SF is the sensitive factor that is 1.00 for C, 1.80 for N and 2.93 for O, respectively.

Supplementary Methods

Chemicals. Tetrahydrofuran (THF, 99.0%), hexane, acetic ester, and diethyl ether were purchased from Kanto Chemicals. Toluene, triisopropyl borate, ammonia water, concentrated HCl, $LiClO_4$, and *n*-butyllithium (1.6 M) were purchased from Wako Chemicals. Copper(I) iodide, 18-crown-6，potassium carbonate, 1,3-dimethyl-3,4,5,6-tetrahydro-2(1H)-pyrimidinone, 1,4-dibromobenzene, and anhydrous magnesium sulfate were purchased from TCI. Tetrakis(triphenylphosphine) palladium(0) and carbazole were purchased from Aldrich. All chromatographic separations were carried out on silica gel (300 mesh).

Instrumental analysis. ^{1}H NMR spectra were recorded on JEOL models JNM-LA400 NMR spectrometers, where chemical shifts (δ in ppm) were determined with a residual proton of the solvent as standard. Fourier transform Infrared (FT IR) spectra were recorded on a JASCO model FT-IR-6100 infrared spectrometer. Matrix-assisted laser desorption ionization time-of-flight mass (MALDI-TOF MS) spectra were recorded on an Applied Biosystems BioSpectrometry model Voyager-DE-STR spectrometer in reflector or linear mode. High-resolution transmission electron microscopy (HR-TEM) images were obtained on a JEOL model JEM-3200. UV-Vis-IR diffuse reflectance spectrum (Kubelka-Munk spectrum) was recorded on a JASCO model V-670 spectrometer equipped with integration sphere model IJN-727. Photoluminescence spectrum was recorded on a JASCO model FP-6600 spectrofluorometer. The absolute quantum yield was determined by standard procedure using an integral sphere JASCO model ILF-533 mounted on the FP-6600 spectrofluorometer. The film thickness was measured on a Veeco Dektak 150 profilometer. Time-resolved fluorescence spectroscopy was recorded on Hamamatsu compact fluorescence lifetime spectrometer Quantaurus–Tau model C11367-11. Kr and nitrogen sorption isotherm measurements were performed by using micromeritics$^{@}$ model 3Flex at 77 K. Spin coating was conducted on a Mikasa spincoater 1H-D7 model at 1500 rpm.

Measurements of fluorescence quantum yield, lifetime and energy transfer efficiency. According to the classic and authoritative textbook in the photophysics field: *Principles of fluorescence spectroscopy, by Joseph R. Lakowicz, 3rd ed. Springer (2006)*, the measurement and calculation of the fluorescence quantum yield, fluorescence lifetime and energy transfer

efficiency are listed as follows.

(1) Fluorescence quantum yield. We utilized a standard integral sphere method implanted in the fluorescence spectrofluorometer. In detail, we measured the fluorescence spectra of the standard background, and calculated its integral area as S_{B0}. Then we measured the fluorescence spectra of the POP films and standard background, and calculated the integral area of the POP films and standard background as S_{POP} and S_{B1}, respectively. The fluorescence quantum yield was calculated by the following equation, $Q = S_{POP} / (S_{B0}–S_{B1})$. In our experiments, the fluorescence quantum yields were automatically calculated by the operation software in the spectrofluorometer.

(2) Fluorescence lifetime. Fluorescence lifetime is defined by $\tau = t / [\ln I(0) - \ln I(t)]$, where $I(0)$ is the initial fluorescence intensity, $I(t)$ is the fluorescence intensity after t period. As for the fluorescence lifetime τ measurements, we utilized Hamamatsu photon-counting device Quantaurus–Tau model C11367-11 and the lifetime was automatically calculated using the operation software implanted in the spectrometer.

(3) Energy transfer efficiency. The energy transfer efficiency is defined as $\Phi_{ENT} = 1 - (I_{DA}/I_D)$, where I_{DA} and I_D are the relative fluorescence intensity of the donor, in the presence and absence of acceptor, respectively. Thus we calculated the energy transfer efficiency by using this equation.

Synthetic procedures

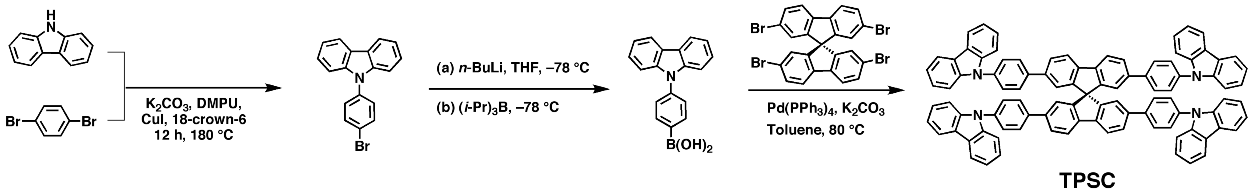

Scheme S1 | The synthesis of TPSC.

9-(4-Bromophenyl)carbazole.[S3] A 1,3-dimethyl-3,4,5,6-tetrahydro-2(1H)-pyrimidinone (DMPU) (20 mL) mixture of CuI (1.14 g, 6 mmol), 18-Crown-6 (0.53 g, 2 mmol), K_2CO_3 (16.6 g, 120 mmol), 1,4-dibromobenzene (14.2 g, 60 mmol) and carbazole (10 g, 60 mmol) was heated at 170 °C for 11 h under argon. After cooling to room temperature, the mixture was quenched with 2 M HCl, and the precipitate was collected and washed with ammonia solution and

deionized water. The grey solid was purified with column chromatography using hexane as an eluent to give 9-(4-bromophenyl)carbazole (11.7 g) as white solid in 74% yield. mp: 145–146 °C. ^{1}H NMR (400 MHz, $CDCl_3$): δ (ppm) = 8.13 (d, 2 H, J = 7.6 Hz), 7.72 (d, 2 H, J = 8.8 Hz), 7.45 (d, 2 H, J = 8.8 Hz), 7.41–7.37 (dt, 4H, J_1 = 6.8 Hz, J_2 = 6.8 Hz), 7.30 (t, 2H, J = 6.6 Hz).

4-Carbazolyl-1-bromophenylboronic acid.[S4] 1.6 M *n*-BuLi in hexane (11.5 mL, 18 mmol) was added dropwise to a solution of 9-(4-bromophenyl)carbazole (4.3 g, 15 mmol) in THF (40 mL) at –78 °C with stirring. After reaction for 1h, triisopropyl borate (32 mL, 18 mmol) was added using a syringe. The mixture was stirred for 1 h, gradually heated to room temperature, and stirred overnight. The clear solution was diluted with ether (100 mL) and washed with deionized water. The organic layer was collected and dried over anhydrous $MgSO_4$. After filtration, the solution was concentrated by using a rotary evaporator. Flash column chromatography of the residue over silica gel with a mixed solvent of hexane and ethyl acetate (9/1 by vol.) as a gradient eluent gave 4-carbazolyl-1-bromophenylboronic acid (3.3 g) as white solid in 87% yield. ^{1}H NMR (400 MHz, $CDCl_3$): δ (ppm) = 8.56 (d, 2H, J = 8.0 Hz), 8.18 (d, 2H, J = 7.6 Hz), 7.80 (d, 2H, J = 8.0 Hz), 7.56 (d, 2H, J = 8.0 Hz), 7.46 (t, 2H, J = 7.2 Hz), 7.33 (t, 2H, J = 7.6 Hz).

2,2',7,7'-Tetra[4-(*N*-carbazolyl)phenyl]-9,9'-spirobifluorene (TPSC).

Tetrakis(triphenylphosphine)palladium (0.034 g, 0.03 mmol) 2,2',7,7'-tetrabromo-9,9'-spirobi[9H-fluorene] (0.517 g, 1.8 mmol), and 4-carbazolyl-1-bromophenylboronic acid (0.189 g, 0.3 mmol) were added to a degassed two-phase mixture of toluene (15 mL) and K_2CO_3 aqueous solution (2 M, 12 mL). The resultant system was vigorously stirred under an argon atmosphere at 85 °C for 24 h. The organic layer was separated and the aqueous phase was extracted with dichloromethane. The organic phases were combined, washed with brine, and dried over anhydrous $MgSO_4$. The solvent was evaporated and the residue was submitted to silica-gel column chromatography with dichloromethane as eluent to give TPSC (0.33 g) as off-white solid in 85% yield. ^{1}H NMR ($CDCl_3$, 400 MHz) δ (ppm) 8.07–8.12 (m, 12H), 7.81 (d, J= 7.60 Hz, 4H), 7.73 (d, J= 7.99 Hz, 8H), 7.54 (d, J= 7.99 Hz, 8H), 7.37 (m, 16H), 7.22–7.27 (m, 12H). ^{13}C NMR ($CDCl_3$, 100 MHz): δ 61.8, 118.1, 119.1, 120.0, 120.4, 121.4, 122.7, 124.7, 126.0, 126.7, 127.3, 128.6. MALDI-TOF MS (calcd for $C_{97}H_{60}N_4$: 1281.54), found m/z = 1281.71 $(M+H)^+$.

TPSC porous polymer powder samples. The TPSC porous polymer powder samples were

synthesized by the chemical oxidation polymerization using ferric chloride as an oxidant. TPSC monomer (80 mg) was dispersed in 30 mL of anhydrous chloroform, and then transferred dropwise to a suspension of ferric chloride (106 mg) in 20 mL of anhydrous chloroform in a 100 mL two-neck flask. The reaction mixture was stirred at room temperature for 24 h under argon atmosphere. Subsequently, 80 mL of methanol was added to the above reaction mixture. The resulting mixture was stirred for 2 h and the precipitate was collected by filtration. The solid was stirred vigorously in concentrated hydrochloric acid solution for 2 h. The suspension was then filtered and washed with water and methanol. Further purification was conducted by using a Soxhlet extractor with methanol and tetrahydrofuran for 24 h, respectively. The powder was dried under vacuum at 110 °C overnight.

Maximum dye loading method. The POP films were immersed into CH_2Cl_2 solutions (20 mL) of coumarin 6 (0.1 M) or nile red (0.1 M) for 12 h at room temperature. The resulting films were repetitively rinsed with CH_2Cl_2 until the solution was clear. The resulting films were dried under vacuum and stocked under Ar in dark. The maximum dye loading contents of in the POP films were determined to be 15.1 and 19.4 mol% for coumarin 6 and nile red, respectively.

Spin-coated film preparations. Coumarin 6 was mixed with TPSC in CH_2Cl_2, which gives a homogenous solution. The solutions with different coumarin 6 content (0.11 – 3.19 mol%) were prepared for tuning the coumarin 6 content and subjected to spin-coat onto the ITO electrode to yield thin films. Because both coumarin 6 and TPSC molecules were completely dissolved in CH_2Cl_2, the spin-coated films are homogenous and the coumarin 6 molecules were homogeneously distributed in the resulting spin-coated films. Therefore, the contents of coumarin 6 in the spin-coated TPSC films are the same as the solutions.

Supplementary References

S1. Li, M., *et al.* Electrochemically deposited organic luminescent films: The effects of deposition parameters on morphologies and luminescent efficiency of films. *J. Phys. Chem. B* **110**, 17784–17789 (2006).

S2. Gu, C., *et al.* Almost completely dedoped electrochemically deposited luminescent films exhibiting excellent LED performance. *Electrochim. Acta* **54**, 7006–7011 (2009).

S3. You, J., Li, G. & Wang, Z. Starburst dendrimers consisting of triphenylamine core and 9-phenylcarbazole-based dendrons: synthesis and properties. *Org. Biomol. Chem.*, **10**, 9481–9490 (2012).

S4. You, J., Li, G., Wang, R., Nie, Q., Wang, Z. & Li, J. Pyrene-cored dendrimer with carbazole derivatives as dendrons: synthesis, properties and application in white light-emitting diode. *Phys. Chem. Chem. Phys.* **13**, 17825–17830 (2011).
